# Study on analog circuit implementation of ReLU Hopfield Neural Network for inequality-constrained optimization problems

**DOI:** 10.3389/fnins.2026.1885313

**Published:** 2026-09-07

**Authors:** Ryosei Okubo, Yuki Mitsuya, Hiroyuki Takahashi

**Affiliations:** 1 School of Engineering, the University of Tokyo, Tokyo, Japan; 2 Kumamoto University, Kumamoto, Japan

**Keywords:** Hopfield Neural Network, inequality-constrained optimization problems, L2 norm, penalty method, ReLU activation function

## Abstract

Though an eminent performance of Artificial Intelligence (AI) is expected to be applied in various technological fields, its high energy consumption is still an issue to be solved for the sustainable implementation of AI into our society. The analog implemented neural networks are promising alternative computation devices with their high-speed convergence and low energy consumption. In this paper, we applied a circuit implemented ReLU Hopfield Neural Network to a mathematical problem with an inequality constraint. After confirming the correspondence between the system dynamics and the search algorithm, we implemented a circuit and observed converged neural circuit outputs that corresponded well to theoretical results and simulations. The objective function value obtained by the proposed analog circuit was within 1.1% relative error of the optimal value.

## 1 Introduction

Although the high performance of AI is both applicable and favorable in various technological fields, there is still a long way to go for sustainable realizations due to its tremendous energy consumption. Nowadays, most computers are built with Von Neumann architecture, featuring a central processing unit that holds a computation unit. This unit retrieves data from and writes to memory with every single process. This data transfer accounts for a significant amount of the energy consumption of the entire computation process (Vogelsang, 2010; Horowitz, 2014). To avoid this data transfer, new technology has been invented: in-memory computing. This technology introduces computation units inside or near the memory. Compared to the conventional Von Neumann architecture, data can be processed inside the memory to avert high-energy transfer. Yet, training an AI model requires frequent re-computation of weights in neural networks; the power usage of training is expected to increase unsustainably.

To address the power consumption problem of AI, analog computation devices have been drawing attention, especially analog circuits. Analog circuits can conduct various calculations by following electrical laws. Since analog circuits show continuous changes of currents and voltages, the calculations are faster compared to digital devices, which run computation by discontinuous clocked changes of currents and voltages. It could be true that analog calculations carry a risk of low computation accuracy. Nonetheless, a proper combination of both analog and digital devices can achieve an adequate load distribution of computation resources and make low-energy-consumption AI possible.

Now, the Hopfield Neural Network, a neural network model advocated by Hopfield in 1982 (Hopfield, 1982), is being spotlighted. This network illustrates how mutually connected neurons change their own states over time and end up in an optimal state. This research provided a new perspective on understanding biological neuron systems as electrical circuits with energy optimization systems. With this network, Hopfield has not only succeeded in storing information within physical systems but also applied it to solving linear optimization problems. Recently, the network, which replaced the sigmoid function with the ReLU function, was reported (Chen et al., 2023a). This network poses an advantage over the original network in terms of simplicity. Though the activation function is replaced by the simple ReLU function, which is represented just by a combination of two linear functions, the rich dynamics of the entire network were conserved, and the network achieved the decreasing number of circuit components by 49%.

Despite the potential applicability of ReLU Hopfield Neural Network, its application to some of the mathematical problems remain unexplored. Among those uninvestigated problems, inequality-constraint optimization problems play crucial roles in modern society in the context of increasing productivity. For instance, to maximize returns of companies, they consider resource allocation problems for their potential markets, or to minimize waiting time for machines in factories, they coordinate their working time by solving coordination problems. Therefore, solving inequality-constrained optimization with analog circuits prospectively widens the application fields of AI. In this paper, we implemented the ReLU Hopfield Neural Network into an analog circuit and solved an optimization problem, namely a single inequality-constraint minimization problem. We installed an inequality-constraint circuit in the analog implemented circuit network to output feedback currents for neurons based on their constraint violations. As an example, we set up a 3-nonnegative-variable quadratic minimization problem with a single inequality constraint. To ensure the convergence of the circuit, the connection matrix, or a matrix form of the minimization problem, was set to be symmetric.

## 2 Materials and methods

### 2.1 Physical computation devices

Utilizing a physical system as a hardware optimization problem solver requires proving an equivalence between the system's dynamics and an optimization algorithm. The equivalence to the gradient method, which uses a gradient of an objective function to determine the search direction, can be written as Equation 1, as proven in Cichocki and Unbehauen (1993).


$$\begin{array}{l} \frac{\text{d}x}{\text{d}t}=-\mu \nabla E\left(x\right)\;\Leftrightarrow \;\frac{\text{d}x_{i}}{\text{d}t}=-\overset{N}{\underset{j=1}{\sum }}\mu_{ij}\frac{\partial E}{\partial x_{j}} \end{array}$$ (1)

where *x* expresses the set of multiple physical quantities at the same time, *E* is the specific energy that is being minimized, or the objective function, and μ is a some positive definite matrix.

### 2.2 Hardware implementation

**Figure 1:**
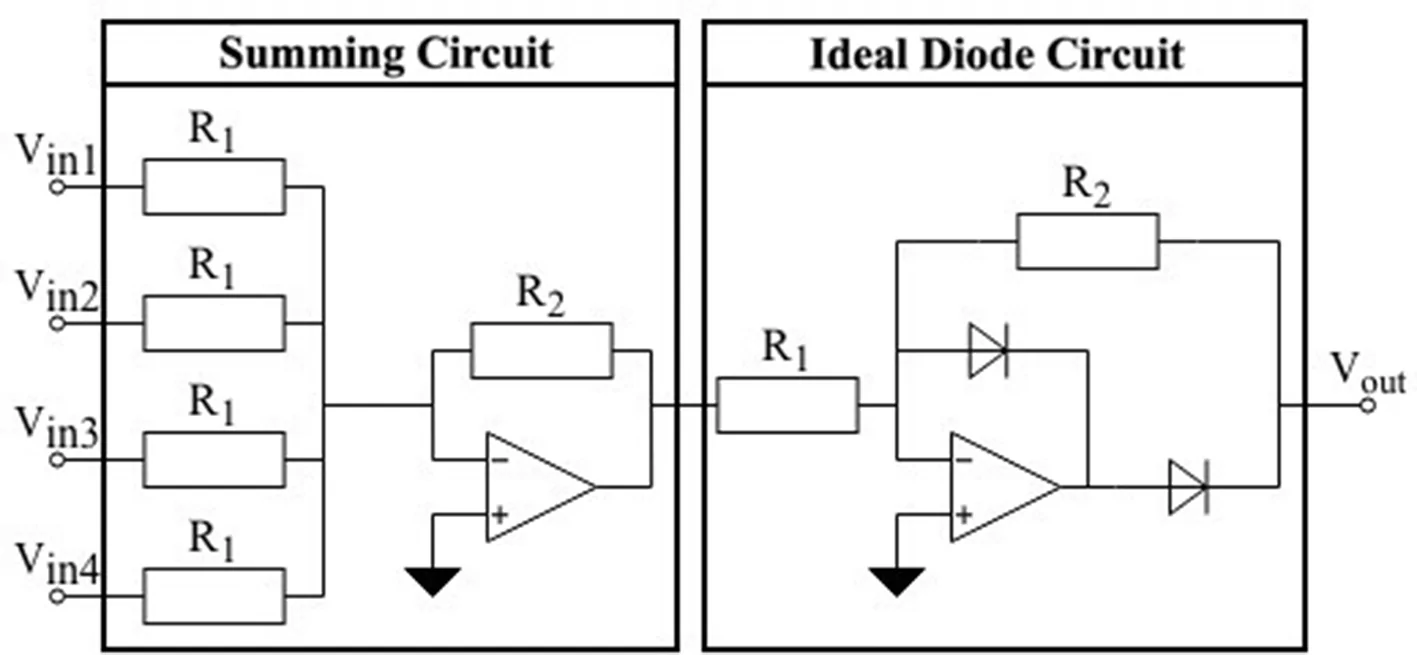
Inequality-constraint circuit.

We constructed an analog circuit of the ReLU Hopfield Neural Network featuring a feedback module: an inequality-constraint circuit. The inequality-constraint circuit was introduced to supply a feedback signal caused by violating the inequality constraint (Cichocki and Unbehauen, 1993), as shown in Figure 1. The circuit adds up all inputs by using the summing circuit and outputs a signal through the ideal diode circuit. We used *L*_2_ norm as the inequality-constrained circuit because of its similarity with the ReLU circuit. Both circuits are built with the ideal diode circuit, and the only difference is the directions of the two diodes. The ReLU Hopfield Neural Network was adapted from Chen et al. (2023a,b) and modified two modules: the connection weight of neurons and the activation function. First, the connection weight was set to symmetry for every mutual connection to ensure the differentiability of the network, and no self-connections were made. Second, we substituted the ReLU function for the reversed ReLU function. This was achieved by adding the inverting amplifier circuit at the end of the original activation function circuit. By inverting the output of the regular ReLU circuit, negative weights can be implemented as positive resistors.

This network's circuit dynamics can be written as a series of differential equations. Suppose the inequality-constraint circuit reacts instantaneously compared to the change of neuron outputs; the output of the inequality-constraint circuit can be considered as the change of bias currents into every neuron. Therefore, using Kirchhoff's current law, the RC charging equation is expressed by Equation 2.


$$\begin{array}{ll} C_{i}\frac{\text{d}u_{i}}{\text{d}t} & =-\left\{\frac{u_{i}}{R_{i}}+\overset{n}{\underset{j=1,j\neq i}{\sum }}\frac{V_{j}}{R_{ij}}+I_{i}+k\times \text{ReLU}\left[r\left(x\right)\right]\right\} \end{array}$$ (2)

*C*_*i*_ is the capacitance of the integration circuit of the neuron *i*, *u*_*i*_ is the internal state represented by voltage, *R*_*i*_ is the resistance of the integration circuit of the neuron *i*, *V*_*i*_ is the output voltage, *R*_*ij*_ is the resistance between neuron *i* and *j*, *I*_*i*_ is the input bias current, *k* is the coefficient representing a strength of ReLU function in the inequality-constraint circuit, and *r*(*x*) is the amount of constraint violation.

Since the correspondence between an internal state and an output is represented by the ReLU function, *V*_*i*_ is equal to *u*_*i*_ as long as *u*_*i*_ is equal to or greater than 0. Therefore, when *u*_*i*_≥0, Equation 2 can be re-written as Equation 3.


$$\begin{array}{ll} C_{i}\frac{\text{d}V_{i}}{\text{d}t} & =-\left\{\frac{V_{i}}{R_{i}}+\overset{n}{\underset{j=1,j\neq i}{\sum }}\frac{V_{j}}{R_{ij}}+I_{i}+k\times \text{ReLU}\left[r\left(x\right)\right]\right\} \end{array}$$ (3)

This network is especially useful for non-negative-variable problems because the circuit ensures the variables' nonnegativeness. The ReLU circuit, realized by the ideal diode circuit, strictly cuts off the voltage at 0 V.

### 2.3 Inequality-constrained optimization problem

As an example, we considered the following inequality-constrained optimization problem (Equation 4), a minimization of a three-non-negative-variable function under a single inequality constraint.


$$\begin{array}{ll} \text{min. } & f\left(x\right)=\frac{1}{2}x^{⊤}Qx+c^{⊤}x \end{array}$$ (4)

$$\begin{array}{l} \text{s.t. }\overset{3}{\underset{i=1}{\sum }}x_{i}\leq 0.5,\;x_{i}\geq 0\;\left(i=1,2,3\right)\\ \text{where }Q=\left(\begin{array}{ccc} \frac{1}{10000} & 1 & 1\\ 1 & \frac{1}{10000} & 1\\ 1 & 1 & \frac{1}{10000} \end{array}\right),c=\left(\begin{array}{c} -1\\ -\frac{1}{2}\\ -\frac{1}{4} \end{array}\right),x=\left(\begin{array}{c} x\\ y\\ z \end{array}\right) \end{array}$$

By using *L*_2_ norm penalty method, this problem can be written as follows:


$$\begin{array}{l} \;\text{min. }f_{\text{penalty}}\left(x\right)=\frac{1}{2}x^{⊤}Qx+c^{⊤}x+\frac{\kappa }{2}\{\text{max}(0,x_{1}+x_{2}\\ \;+x_{3}-0.5){\}}^{2}\\ \;\text{s.t. }x_{i}\geq 0\;\left(i=1,2,3\right)\\ \text{where }Q=\left(\begin{array}{ccc} \frac{1}{10000} & 1 & 1\\ 1 & \frac{1}{10000} & 1\\ 1 & 1 & \frac{1}{10000} \end{array}\right),c=\left(\begin{array}{c} -1\\ -\frac{1}{2}\\ -\frac{1}{4} \end{array}\right),x=\left(\begin{array}{c} x_{1}\\ x_{2}\\ x_{3} \end{array}\right),\\ \kappa =const. \end{array}$$

κ is the penalty coefficient. The penalty term regulates the bias currents into neurons by changing its output depending on the constraint violation. The penalty term outputs 0 while the constraint is observed, but once outputs from neurons violate the constraint, the penalty term outputs positive values to suppress the neurons. Since *Q* is a symmetric matrix, the derivative of *f*_penalty_(***x***) is


$$\begin{array}{l} \frac{\text{d}f_{\text{penalty}}\left(x\right)}{\text{d}x_{i}}=Q_{ii}x_{i}+\underset{j\neq i}{\sum }Q_{ij}x_{j}+c_{i}+\kappa \times \text{max}\left(0,\overset{3}{\underset{i=1}{\sum }}x_{i}-0.5\right) \end{array}$$ (5)

By comparing Equation 5 with Equation 3, we obtain the following correspondence:


$$\begin{array}{l} V_{i}\propto x_{i},\;\frac{1}{R_{i}}\propto Q_{ii},\;\frac{1}{R_{ij}}\propto Q_{ij},\;I_{i}\propto c_{i},\;k\propto \kappa \end{array}$$

**Figure 2:**
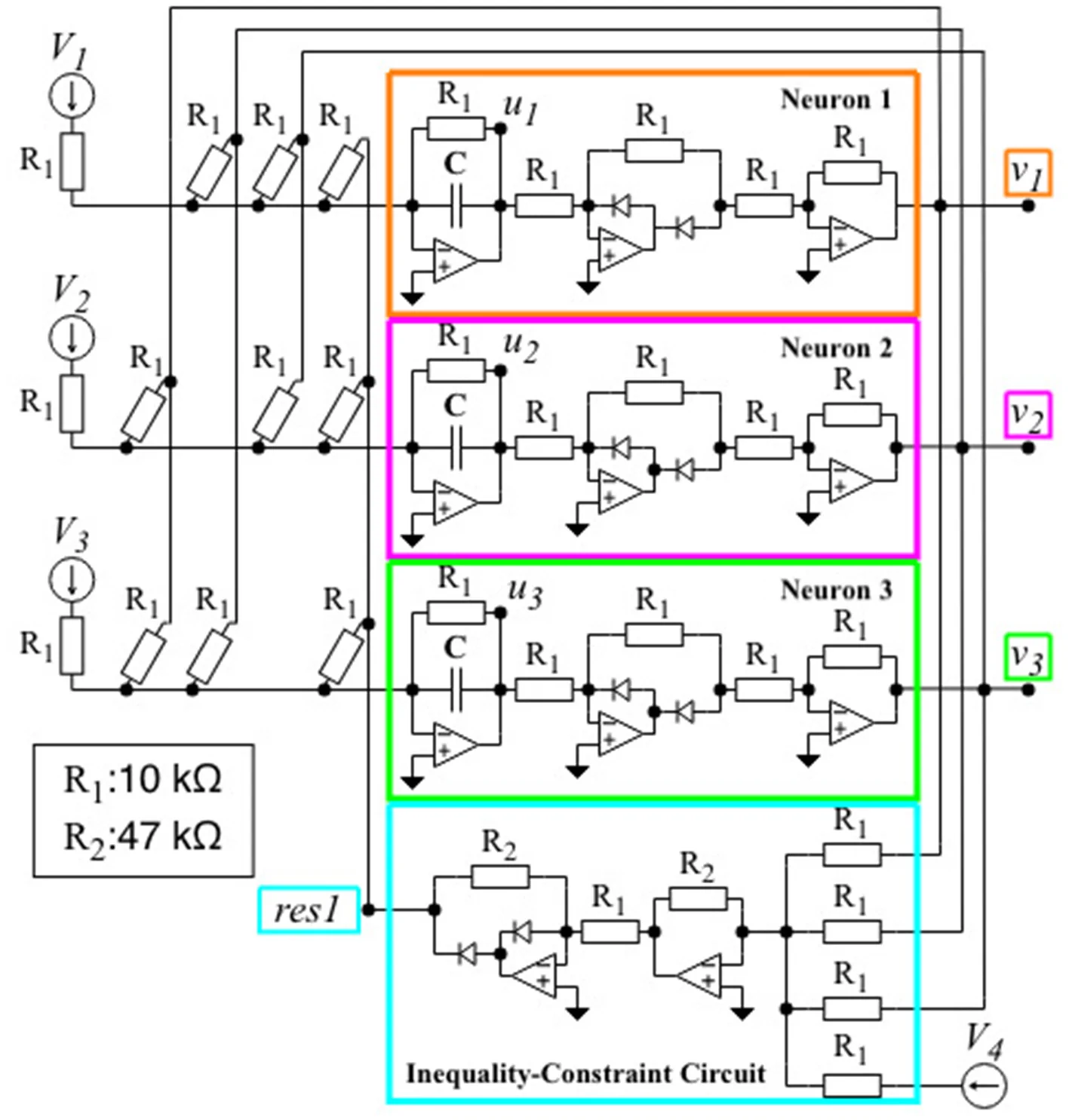
Circuit schematics.

Thus, the circuit parameters are decided as shown in Figure 2. *c*_*i*_ values are given by supply voltages of *V*_*i*_. Voltages of *v*_*i*_ represents the computation result of variable *x*_*i*_. The penalty coefficient κ was set to 22.09, the gain of the inequality-constraint circuit.

By solving the problem algebraically under κ = 22.09, the optimal solution is:


$$\begin{array}{l} x^{*}=\left(\frac{1}{2}+\frac{19999}{20000\kappa +2},0,0\right){|}_{\kappa =22.09}\approx \left(0.545,0.000,0.000\right) \end{array}$$

This network's dynamics are equivalent to the gradient method, and the network executes a minimization of a specific energy, corresponding to *f*_penalty_, through changes in output voltages. If the internal states of each neuron remains above 0, the following relationships hold true.


$$\begin{array}{l} C_{i}\frac{\text{d}u_{i}}{\text{d}t}\propto -\frac{\partial f_{\text{penalty}}}{\partial x_{i}}\;\Leftrightarrow \;\frac{\text{d}u_{i}}{\text{d}t}=-\frac{a_{i}}{C_{i}}\frac{\partial f_{\text{penalty}}}{\partial x_{i}}\;\left(a_{i}=const.\right) \end{array}$$

With a chain rule, we finally get:


$$\begin{array}{l} \frac{\text{d}x_{i}}{\text{d}t}=-\overset{3}{\underset{j=1}{\sum }}\mu_{ij}\frac{\partial f_{\text{penalty}}}{\partial x_{i}},\;\mu =\left(\begin{array}{ccc} \frac{a_{1}}{C_{1}} & 0 & 0\\ 0 & \frac{a_{2}}{C_{2}} & 0\\ 0 & 0 & \frac{a_{3}}{C_{3}} \end{array}\right) \end{array}$$

This satisfies the gradient method relationship (Equation 1), and the system searches for the optimal solution. Indeed, using the gradient methods could end up in local solutions, but the problem we tackled does not have either relative minimum values or saddle points inside and near the feasible regions. Thus, the network converges to the state that represents the optimal solution. Moreover, the negative voltages of the internal state may undermine the search algorithm by breaking the *u*_*i*_ = *V*_*i*_ relationships. However, this does not affect the solving process due to a clamp by the ReLU function. Once the neuron's internal state is lower than 0, its output is kept 0. The turned-off neuron does not influence other neurons' dynamics. Therefore, if the network does not fall into a relative minimum or saddle points, the dynamics of the network maintain the search algorithm.

## 3 Results

We conducted experiments on two different platforms: a simulation on a computer and a printed circuit board.

### 3.1 Simulation

First, we simulated the analog implemented circuit on LTspice^Ⓡ^ ver. 17.2.4. with a transient analysis. The simulation duration was 2 ms and the time step was 0.00047 ms. In the circuit, resistors and capacitors have precise resistances and capacitance, and diodes and op-amps had their own property characteristics. The time constant is τ = 10000Ω×47 nF = 0.47 ms.

**Figure 3:**
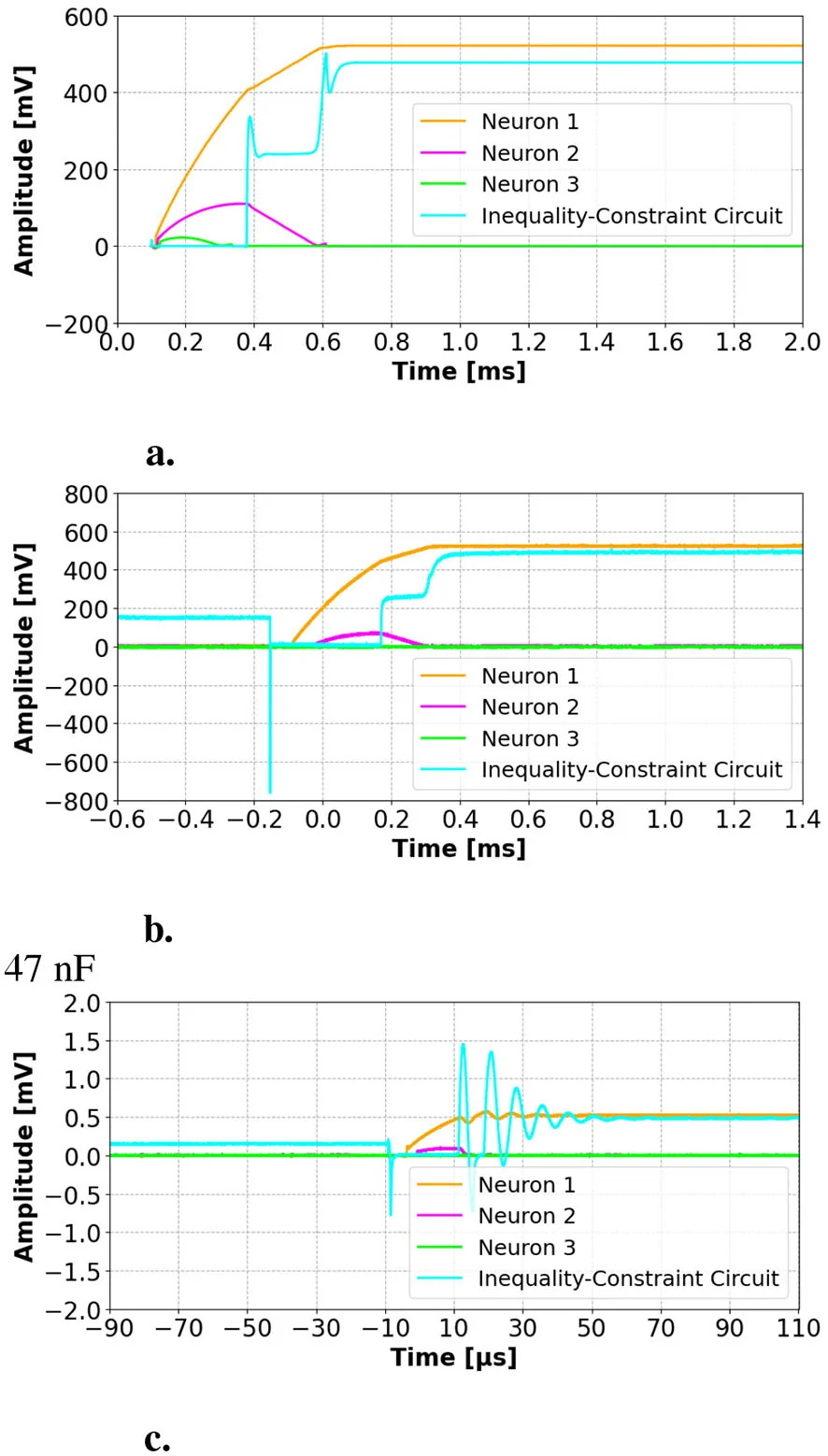
Voltage changes of each node over time. **(a)** The simulation result, **(b)** the experiment result with capacitance of 47 nF, and **(c)** the experiment result with capacitance of 2.2 nF.

The voltage change over time is shown in Figure 3a. The voltage of each output at 2 ms is summarized in Table 1.

**Table 1:** Simulated voltage at each node at 2 ms after the input.

Node	Voltage [V]
*v*_1_	0.522
*v*_2_	0.000
*v*_3_	0.000
*res*1	0.478

### 3.2 Experiment

After conducting a simulation on a computer, we carried out experiments with actual circuits by designing a printed circuit board and monitored the changes in voltage at neurons over time. To investigate the effects of time constants, we made it possible for capacitors to be replaced easily by introducing pins on the circuit board. Though the resistance of the integrating circuit at the input of each neuron could also alter the time constants, changing them alters the connection matrix, which leads to a contradiction between the problem and the network dynamics.

The experimental equipment consists of a printed circuit board, an external input device, a power supply, and an oscilloscope. We attached a power supply for op-amps and a Field Programmable Gate Array to input bias currents. All bias currents were provided from a single FPGA to ensure simultaneity; the system is vulnerable to local minima, and the lag between input currents may lead to an unexpected convergence.

Figure 3b shows the time dynamics of voltages of each node. Note that the graph's 0 seconds does not represent the time of input. We observed the similar waveform compared to the simulation result. Also, we sampled the voltages 1250 times between 500 and 600 ms. Table 2 shows the average voltage and standard errors of every node.

**Table 2:** Average voltages and standard errors observed at nodes when the capacitor is set to 47 nF.

Node	Average voltage [V]	Standard error [V]
*v*_1_	0.523	4.61 × 10^−5^
*v*_2_	0.001	6.76 × 10^−5^
*v*_3_	−0.002	4.35 × 10^−5^
*res*1	0.488	6.96 × 10^−5^

The sample size was *n* = 1250.

Additionally, to research the behavior under high-speed conditions, we replaced all capacitors with 2.2 nF capacitances and studied their output changes. Figure 3c shows the waveforms of this case. Besides, we sampled the voltages 1,249 times between 100 and 110 μs. Table 3 shows the average voltage and standard errors of every node. We observed an oscillation in the inequality-constraint circuit output.

**Table 3:** Average voltages and standard errors observed at nodes when the capacitor is set to 2.2 nF.

Node	Average voltage [V]	Standard error [V]
*v*_1_	0.521	8.36 × 10^−5^
*v*_2_	0.001	9.48 × 10^−5^
*v*_3_	−0.002	7.98 × 10^−5^
*res*1	0.485	9.39 × 10^−5^

The sample size was *n* = 1249.

## 4 Discussion

### 4.1 Optimization performance

In the experiment, all the circuit components functioned as expected. One of the most important factors of the experiment is the functional precision of op-amps, because most of the calculation mechanisms involve the op-amps, such as the integration circuits, the summing circuits, the amplifier circuit, and the ideal diode circuits. The desired functions of op-amps can be understood by their output voltages. Since the voltage of the entire circuit was less than ±15 V, the operating voltage of the op-amps, the components functioned as expected.

The theoretical values, simulation values, and experimental values are summarized in Table 4. “Original Problem” refers to the original optimization problem, and “Penalty-form Problem” refers to the optimization problem with *L*_2_ norm penalty method. The standard errors are within 0.0002 V range. For variable *x*_1_ and function value *f*(*x*), whose theoretical values are not zero, relative errors were 4.4% and 1.1%, calculated by $\frac{\left|\left(Actual\;value\right)-\left(Theoretical\;value\right)\right|}{Theoretical\;value}$.

**Table 4:** Comparison of Theoretical, Simulated, and Experimental values.

Condition	*x*_1_	*x*_2_	*x*_3_	*f*(*x*)
Original problem	0.5	0	0	−0.500
Penalty-form problem	0.545	0	0	−0.523
Simulation: 47 nF	0.522	0.000	0.000	–
Experiment: 47 nF	0.523 ± 4.61 × 10^−5^	0.001 ± 6.76 × 10^−5^	−0.002 ± 4.35 × 10^−5^	–
Experiment: 2.2 nF	0.521 ± 8.36 × 10^−5^	0.001 ± 9.48 × 10^−5^	−0.002 ± 7.98 × 10^−5^	−0.517

Experiment values are shown in (Average ± Standard Error). Sampling sizes are *n* = 1, 250 for 47 nF and *n* = 1, 249 for 2.2 nF, respectively.

Through the experiments, the implemented neural network circuit showed a 1.1% function value error compared to the theoretical penalty-form problem function value, which is closer to the original minimization problem. The variables avoided unexpected stationary points and successfully converged to values near the expected solutions.

The errors may be caused by the following two reasons: components' 5% tolerances or unintentional backflows of currents into the inequality-constraint circuit. All the circuit components possess a manufacturing tolerance of 5% for their property characteristics. Additionally, the backflow into the inequality-constraint circuit may cause the error. Originally, the circuit was designed under the circumstance where all output currents from the neurons and the inequality-constraint circuit flow into neuron inputs. However, the inequality-constraint circuit could have absorbed the currents that were supposed to enter the neuron inputs. This can be understood by the non-zero voltages of each node at the beginning of the experiments.

### 4.2 Further extensions and limitations

In this section, we will address an extension method for larger network implementations and its underlying limitations.

Adaptability to different optimization problems can be achieved by introducing variable resistors. In this study, we used fixed-value resistors and encoded a single corresponding optimization problem. To solve a different problem, the resistor values must be modified. By using variable resistors, we can reuse the boards for different problems. To handle many neurons and constraints, a digital potentiometer-based implementation would be useful, since the resistor values can be controlled digitally from software.

The scalability of this analog implementation is another important issue to discuss. Theoretically, this implementation scheme can deal with more variables and inequality constraints by increasing the number of neurons and constraint modules. By implementing the neurons and constraint circuits as modular printed circuit boards, the system could flexibly handle larger problems. However, the bottleneck lies in the number of connections between neurons, which scales as *O*(*n*^2^) for *n* neurons. The rapidly growing number of connections increases circuit complexity and makes wiring difficult. A conceivable solution is to exploit a possible sparse representation of *Q* in Equation 4 and of the inequality constraints, which correspond to the connections between the neurons and the constraint circuit.

The variability in component characteristics should also be considered and investigated for practical applications. Although high-precision components have lower variability, they are more expensive. A study should be conducted to experimentally investigate how the variability of each circuit component contributes to the final result. Another important effect is temperature variation. Heavier computations with a large number of neurons would raise the board temperature and hence change the electrical properties of each component. Environmental heat is another source of temperature rise. Investigating this temperature effect is also left as future work.

## 5 Conclusion

Exploring the applications of the ReLU Hopfield Neural Network is invaluable because of its low-energy calculations. As opposed to digital calculation devices, analog-based calculation devices, including the ReLU Hopfield Neural Network, can conduct computations with lower energy by following the electrical laws.

In this paper, we succeeded in solving a quadratic programming problem through the analog circuit implemented ReLU Hopfield Neural Network. We also confirmed that the implemented network is possible to compute with higher time constant conditions.

## Data Availability

The original contributions presented in the study are included in the article/supplementary material, further inquiries can be directed to the corresponding author/s.
